# Mammographic density changes in surgical weight loss-an indication for personalized screening

**DOI:** 10.1186/s12880-017-0242-4

**Published:** 2018-05-09

**Authors:** Natalia Partain, Ali Mokdad, Nancy Puzziferri, Jessica Porembka, Stephen Seiler, Alana Christie, Deborah Farr, Aeisha Rivers, A. Marilyn Leitch, Rachel Wooldridge, James Huth, Roshni Rao

**Affiliations:** 1 Department of Surgery, Division of Surgical Oncology, University of Texas Southwestern Medical Center, 5323 Harry Hines Blvd., Dallas, TX 75390-8548 USA; 2 Department of Surgery, Division of Gastrointestinal and Endocrine Surgery, University of Texas Southwestern Medical Center, 5323 Harry Hines Blvd., Dallas, TX 75390-8548 USA; 3 Department of Radiology, University of Texas Southwestern Medical Center, 5323 Harry Hines Blvd., Dallas, TX 75390-8548 USA; 4 Division of Breast Surgery, Columbia University Medical Center/New York Presbyterian, 161 Fort Washington Ave 10th floor, New York, NY 10032 USA

**Keywords:** Mammographic density, Breast cancer, Surgical weight loss, Bariatric surgery

## Abstract

**Background:**

Obesity and high radiologic breast density independently increase breast cancer risk. We evaluated the effect of surgical weight loss on mammographic density (MD).

**Methods:**

Patients undergoing bariatric surgery and screening mammography (MG) were identified, data regarding demographics, comorbidities, calculated and genetic breast cancer risk was collected. Patients had a MG before and after surgery. Fellowship-trained breast radiologists assigned Breast Imaging Reporting and Data System density categories.

**Results:**

Patients underwent sleeve gastrectomy (*n* = 56) or gastric bypass (*n* = 7), 78% had hypertension, 48% had diabetes. Four had deleterious BRCA mutations, four were calculated high risk. Mean weight loss = 28.7 kg. Mean initial BMI = 44.3 kg/m^2^ (range:33–77), final BMI = 33.6 kg/m^2^ (range:20–62;*p* < 0.01). Density was unchanged in 53, decreased in 1, increased in 9. Of these 9(14%), 5 changed from almost entirely fatty to scattered MD, and 4 changed from scattered MD to heterogeneously dense. Mean weight loss of the 9 with increased MD was greater than the cohort (37.7vs.28.7 kg;*p* < 0.01).

**Conclusions:**

Surgical weight loss increased MD in 14%. Increased MD masks malignancies, patients may benefit from additional screening based on calculated risk assessments that include MD.

## Background

### Breast density

**Fig. 1:**
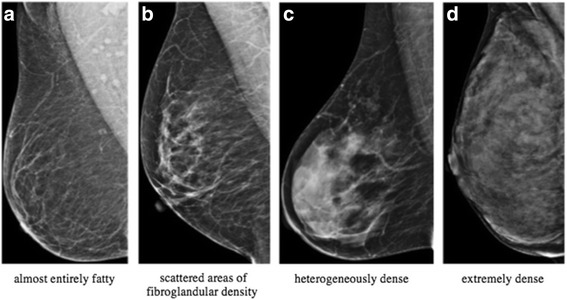
Examples of BI-RADS breast composition categories of breast density in increasing order of density from left to right (panels **a**-**d**)

Mammographic breast density is caused by a mixture of stromal, glandular, and adipose tissue. Radiologically dense tissue, which is primarily composed of stromal and epithelial components, typically appears radiopaque on mammography, while adipose tissue appears dark and radiologically lucent [1, 2]. The American College of Radiology (ACR) created a standardized lexicon in the Breast Imaging-Reporting and Data System (BI-RADS) Atlas for reporting overall breast composition on mammograms. The overall mammographic breast composition is divided into four categories: (a) almost entirely fatty, (b) scattered areas of fibroglandular density, (c) heterogeneously dense and (d) extremely dense [3, 4] (Fig. 1). Overall breast tissue composition that is considered mammographically dense is categorized as either heterogeneously dense or extremely dense. Increased tissue density is known to obscure breast cancer and can significantly decrease the sensitivity of mammography secondary to a decrease in contrast resolution between a radiopaque malignancy and dense breast tissue [4]. In addition, studies reveal a potential correlation between breast cancer and increased breast density [5–7]. Women with mammographically dense breasts have an increased relative risk of developing breast cancer 4 to 6 times that of low breast density women; this increased risk remains even after adjusting for confounding variables [4, 8–11]. This relative risk elevation is larger than the risk associated with family history or any other reproductive risk factors [4].

### Obesity and breast cancer

Obesity, defined by an elevated body mass index, occurs as a consequence of excess adipose tissue [12]. Adipose tissue, the largest endocrine organ in the body, plays a role in energy homeostasis. Unchecked hyperadiposity may lead to metabolic disorders, altered production of steroid hormones (estrogen) and adipokines, and chronic subclinical inflammation [13, 14]. All of these pathophysiologic changes have been associated with cancer development, especially in estrogen-dependent postmenopausal breast cancer [15]. The main source of estrogen in postmenopausal women is adipose tissue. At the molecular level, excess white adipose tissue leads to endoplasmic reticulum stress, tissue fibrosis, and local hypoxia. This, in turn, triggers a vicious cycle of inflammation to include adipocyte cell death and recruitment of macrophages, and increased levels of aromatase, the rate-limiting enzyme in estrogen production. Weight reduction, by lowering systemic and local estrogen production, is thus important in cancer prevention and treatment [13, 14]. Surgically-induced weight loss appears to decrease the risks of endometrial, breast, and ovarian cancers [16, 17]. Bariatric surgery has also been shown to improve outcomes in colorectal cancer [18]. Additionally, a study evaluating the post-menopausal symptoms of vaginal dryness and flushing demonstrated decreased rates of symptomatology after bariatric surgery, potentially underscoring estrogen associated changes due to surgically-induced weight loss [19].

### Bariatric surgery and breast density

Mammographic breast density can be inversely related to body weight but is associated with increased breast cancer risk. Paradoxically, obesity is also associated with increased breast cancer risk [5, 20]. Studies are underway to evaluate if dietary changes and physical activity are able to reduce mammographic breast density in postmenopausal women [21]. Some studies have shown that density increases with weight loss [22–24]. Therefore, due to the lack of clarity regarding the impact of weight loss on density, this study evaluates the effect of weight loss on mammographic breast density.

## Methods

Approval for this study was obtained from the Institutional Review Board (STU 032015–088) at the University of Texas Southwestern Medical Center (UTSW). Initial medical record review queried for female bariatric surgery patients who were ≥40 years of age and therefore would have been advised to undergo screening mammography. These 700 records were cross-referenced to identify patients who had undergone weight loss surgery and mammographic screening within the UTSW medical center from 2008 to 2015. Data collected included demographics, breast cancer risk factors, co-morbidities, development of cancer, and genetic mutation status. Three-hundred patients had screening mammograms. Patients were excluded if they had a history of breast cancer prior to bariatric surgery or did not have digital imaging available within the UTSW system. Sixty-three patients included had a mammogram prior to and at least 1 year after surgery. The mammograms of included patients were then retrieved from the digital imaging archive. The mediolateral oblique view was selected as the image to review for assignment of overall breast composition, as this view includes more fibroglandular tissue than does the craniocaudal view, especially in the upper outer quadrant, where most cancers develop [25]. All mammogram images (void of any personal identifiers, information overlays, or dates of examinations) were reviewed by two board certified radiologists who are fellowship trained in breast imaging. The radiologists were blinded to all information about the participants including identification number and the temporal sequence of the mammograms. The radiologists then performed an independent clinical assessment of overall mammographic breast composition using the ACR BI-RADS Atlas 5th Edition. This approach to categorizing density is currently the most commonly used approach in the United States, and subsequently the most clinically applicable [26]. Statistical analysis was carried out using chi-square and paired t-test, SAS 9.4, with a *p* value less than <0.05 considered statistically significant. Any change in mammographic density before and after weight loss surgery was evaluated.

## Results

### Patient characteristics

Sixty-three patients were identified who met eligibility criteria. Mean age at the time of bariatric surgery was 51.7 years (Table 1). The majority of women were Caucasian (65.1%), postmenopausal (77.8%), and had an average of 4 comorbidities with the most common being hypertension, hyperlipidemia and reflux. The majority of the mammograms (*n* = 48, 76%) were performed for routine screening. Patients underwent a variety of bariatric operations with 73% (*n* = 46) undergoing sleeve gastrectomy, 16% (*n* = 10) had a prior laparoscopic adjustable gastric band placed with subsequent conversion to a sleeve gastrectomy, and 11% (*n* = 7) underwent a gastric bypass procedure. Four patients were known BRCA mutation carriers.

**Table 1:** Patient Characteristics

	*n* (%)
Age (mean ± std)	51.7 ± 8.2
Race	
Black	22 (34.9)
White	41 (65.1)
Reason for mammogram	
High risk	14 (22.2)
Prior benign	1 (1.6)
Screening	48 (76.2)
Menopause status	
Pre-menopausal	14 (22.2)
Post-menopausal	49 (77.8)
Morbidities (mean ± std)	4.0 ± 1.5
Diabetes	32 (50.8)
Hypertension	49 (77.8)
Sleep apnea	30 (47.6)
Reflux	44 (69.8)
Hyperlipidemia	40 (63.5)
Osteoarthritis	36 (57.1)
Heart disease	10 (15.9)
Fatty liver	8 (12.7)
Procedure	
Gastric bypass	7 (11.1)
Gastric sleeve	46 (73.0)
Lap band to sleeve	10 (15.9)

*Std* standard deviation

### Changes in weight and BI-RADS density

**Fig. 2:**
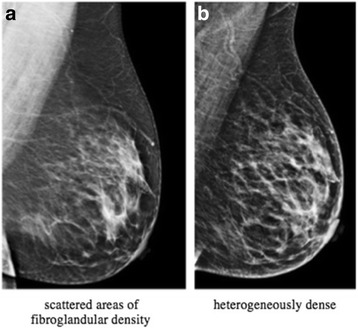
Initial (**a**) and final (**b**) mammograms of a study patient who underwent weight loss surgery showing change from scattered areas of fibroglandular density to heterogeneously dense

As expected, there was a statistically significant decrease in weight and Body Mass Index (BMI) after bariatric surgery. Average weight loss was 28.7 kg (95% CI: 25.1–33.3; *p* < 0.0001) and BMI decreased by 10.6 kg/m^2^ (95% CI: 8.9–12.3; p < 0.0001) (Table 2). Both breast imagers were “blinded” and assigned independently the same density to each mammographic film, showing accuracy and inter-observer agreement. For the change in BI-RADS density, patients who started out as scattered areas of fibroglandular density or heterogeneously dense tended to stay the same after weight loss surgery, while about half the patients that were almost entirely fatty density increased to scattered areas of fibroglandular density (Table 3) (Fig. 2). The mean time from initial mammogram to surgery, surgery to final mammogram, and initial to final mammogram was 55.6, 85.2, 140.8 weeks, respectively. Only one patient, who had an initial and final mammographic breast composition of scattered areas of fibroglandular density, developed breast cancer a year and 8 months after surgery.

**Table 2:** Comparison between preoperative and postoperatively BMI and weight values

	Before Surgery (mean/median)	After Surgery (mean/median)	*P*-value
BMI (kg/m^2^)	44.3 / 42	33.7 / 32	0.0001
Weight (kg)	119.4 / 108.2	90.7 / 85.4	0.0001

*BMI* body mass index

**Table 3:** Mammographic breast composition before and after weight loss surgery

			Initial BIRADS density	
	Almost entirely fatty	Scattered fibroglandular	Heterogeneously dense	*p*
Final BI-RADS density				<0.0001
Almost entirely fatty	6 (54.5)	0 (0)	0 (0)	
Scattered fibroglandular	5 (45.5)	40 (90.9)	1 (12.5)	
Heterogeneously dense	0 (0)	4 (9.1)	7 (87.5)

## Discussion

In our study, significant and rapid surgical weight loss appeared to impact mammographic density. As previously mentioned, increased mammographic density has been associated with greater stromal area and less adipose tissue; however there is conflicting evidence regarding changes in the epithelial areas [2, 27–29].

### Biology of breast density

The biologic and molecular contributions to breast density are unclear. Research has focused on studying cellular proliferation markers such as Ki-67 with inconsistent results. Gabrielson et al., [2] studied the histo-biologic composition of normal breast tissue in relation to mammographic density by obtaining ultrasound-guided breast biopsies in 160 healthy women. There was no association between high mammographic breast density and increased epithelial levels of Ki-67 and the expression of epithelial estrogen or progesterone receptors. Epithelial Ki-67 was associated with a greater epithelial proportion, and epithelial progesterone receptors were associated with greater stromal and lower adipose proportions [2]. It is believed that cumulative exposure to various hormones and growth factors may mediate breast tissue changes associated with increased breast cancer risk. The International Breast Cancer Intervention Study demonstrated that for women with increased breast cancer risk, initiating chemoprevention with tamoxifen can lower mammographic breast density. Women were given 20 mg of tamoxifen once daily versus placebo for 5 years. Forty-six percent of patients taking tamoxifen had at least a 10% or more decline in breast density. These women also had a 63% lower breast cancer incidence compared to placebo or to those women who experienced a less than 10% reduction in mammographic density [7]. Breast density changes in postmenopausal women similar to our cohort are more limited, and there is a paucity of data regarding usage of aromatase inhibitors when density changes have occurred [8]. The Alberta Physical Activity and Breast Cancer Prevention Trial examined aerobic exercise and breast density in postmenopausal women. This study randomized women, who had been postmenopausal for at least 24 months, sedentary and not on any hormone replacement therapy, to a one-year aerobic exercise intervention in comparison to usual sedentary lifestyle. Exercisers had a significant decrease in the non-dense volume that correlated to a decrease in percent body fat compared to the control group (2% versus 0.2%, *p* = 0.001). Changes in the amount of fibroglandular tissue in the breast were not affected by exercise [30]. Our study similarly demonstrates that women with breast tissue composed mainly of scattered areas of fibroglandular density or heterogeneously dense tissue do not have significant density changes, even with large amounts of surgical weight loss. In breasts mainly composed of almost entirely fatty density, the density seemed to increase with weight loss, potentially as a result of an overall decrease in intervening adipose tissue within the breast.

### Implications of increasing breast density and the paradox of surgical weight loss

With increases in breast tissue density, the sensitivity of mammography decreases. The addition of breast density to the Gail model, which is the most widely used method of predicting breast cancer in individuals, showed increased predictive accuracy with increasing density correlating with increased risk [4, 31, 32]. It is unclear then, how the increasing mammographic breast density seen with surgical weight loss correlates with the known overall reduction in breast cancer risk after bariatric surgery. In the United States, there continues to be significant debate and discordance between physician groups, advocacy agencies, and government panels regarding optimal mammographic screening practices. As the risk factors for breast cancer are more finely defined, potentially using results from studies such as this one, personalized screening practice may have more widespread support.

Given the increasing density seen in some of the patients in this study, it may be useful to perform breast cancer risk calculations on patients having undergone bariatric surgery, in order to potentially allow personalized screening practices that may include tomosynthesis or breast magnetic resonance imaging depending on risk. In patients who had complete medical records and a change from scattered areas of fibroglandular density to heterogeneously dense breasts, the calculated 5-year risk of breast cancer increased when density was included in the calculations. With the addition of larger numbers of patients, more specific nomograms that quantify the additive risk of changes in density could be developed.

Limitations to our study include its retrospective nature, small sample size, and reliability of a qualitative analysis of mammograms as opposed to quantitative analysis with an automated computer software.

Future studies could include a prospective collection of breast tissue biopsy before and after weight loss surgery in order to compare histology to mammographic density and identify mechanisms for this change in density.

## Conclusions

Surgical weight loss in postmenopausal women appears to induce heterogeneous changes in mammographic breast density composition. Some patients demonstrated increased density, which could decrease the sensitivity of mammography. These patients may benefit from a more personalized approach to screening depending on their individual breast cancer risk.

## Abbreviations

ACR: American College of Radiology
BI-RADS: Breast Imaging-Reporting and Data System
BMI: Body Mass Index
MD: Mammographic density
MG: Mammography
UTSW: University of Texas Southwestern Medical Center

## References

[1] Boyd NF, Lockwood GA, Martin LJ (1998). Mammographic densities and breast cancer risk. Breast Dis.

[2] Gabrielson M, Chiesa F, Paulsson J (2016). Amount of stroma is associated with mammographic density and stromal expression of oestrogen receptor in normal breast tissues. Breast Cancer Res Treat.

[3] Sickles EA, D'Orsi CJ, Bassett LW (2013). Mammography*.* ACR BI-RADS® atlas, breast imaging reporting and data system.

[4] Boyd NF, Martin LJ, Yaffe MJ, Minkin S (2011). Mammographic density and breast cancer risk: current understanding and future prospects. Breast Cancer Res.

[5] Martin LJ, Boyd NF (2008). Mammographic density. Potential mechanisms of breast cancer risk associated with mammographic density: hypotheses based on epidemiological evidence. Breast Cancer Res.

[6] McCormack VA, dos Santos Silva I (2006). Breast density and parenchymal patterns as markers of breast cancer risk: a meta-analysis. Cancer Epidemiol Biomark Prev.

[7] Cuzick J, Warwick J, Pinney E (2011). Tamoxifen-induced reduction in mammographic density and breast cancer risk reduction: a nested case-control study. J Natl Cancer Inst.

[8] Mullooly M, Pfeiffer RM, Nyante SJ (2016). Mammographic density as a biosensor of Tamoxifen effectiveness in adjuvant endocrine treatment of breast cancer: opportunities and implications. J Clin Oncol.

[9] Huo CW, Chew GL, Britt KL (2014). Mammographic density-a review on the current understanding of its association with breast cancer. Breast Cancer Res Treat.

[10] Boyd NF, Byng JW, Jong RA (1995). Quantitative classification of mammographic densities and breast cancer risk: results from the Canadian National Breast Screening Study. J Natl Cancer Inst.

[11] Boyd NF, Guo H, Martin LJ (2007). Mammographic density and the risk and detection of breast cancer. N Engl J Med.

[12] Rutkowski JM, Stern JH, Scherer PE (2015). The cell biology of fat expansion. J Cell Biol.

[13] Zahid H, Simpson ER, Brown KA (2016). Inflammation, dysregulated metabolism and aromatase in obesity and breast cancer. Curr Opin Pharmacol.

[14] Iyengar NM, Gucalp A, Dannenberg AJ, Hudis CA (2016). Obesity and cancer mechanisms: tumor microenvironment and inflammation. J Clin Oncol.

[15] Keum N, Greenwood DC, Lee DH, et al. Adult weight gain and adiposity-related cancers: a dose-response meta-analysis of prospective observational studies. J Natl Cancer Inst. 2015;107(2):1–14. 10.1093/jnci/djv088 25757865

[16] Christou NV, Lieberman M, Sampalis F, Sampalis JS (2008). Bariatric surgery reduces cancer risk in morbidly obese patients. Surg Obes Relat Dis.

[17] McCawley GM, Ferriss JS, Geffel D (2009). Cancer in obese women: potential protective impact of bariatric surgery. J Am Coll Surg.

[18] Hussan H, Stanich PP, Gray DM, et al. Prior Bariatric Surgery Is Linked to Improved Colorectal Cancer Surgery Outcomes and Costs: A Propensity-Matched Analysis. Obes Surg. 2016;4:1047–55. 10.1007/s11695-016-2421-8 27770262

[19] Goughnour SL, Thurston RC, Althouse AD (2016). Assessment of hot flushes and vaginal dryness among obese women undergoing bariatric surgery. Climacteric.

[20] Boyd NF, Martin LJ, Sun L (2006). Body size, mammographic density, and breast cancer risk. Cancer Epidemiol Biomark Prev.

[21] Masala G, Assedi M, Caini S (2014). The DAMA trial: a diet and physical activity intervention trial to reduce mammographic breast density in postmenopausal women in Tuscany, Italy. Study protocol and baseline characteristics. Tumori.

[22] Hart V, Reeves KW, Sturgeon SR (2015). The effect of change in body mass index on volumetric measures of mammographic density. Cancer Epidemiol Biomark Prev.

[23] Mokhtari TE, Rosas US, Downey JR, et al. Mammography before and after bariatric surgery. Surg Obes Relat Dis. 2016;13:451–56. 10.1016/j.soard.2016.10.021 27986574

[24] Vohra NA, Kachare SD, Vos P, et al. The Short-Term Effect of Weight Loss Surgery on Volumetric Breast Density and Fibroglandular Volume. Obes Surg. 2016;27(4):1013–23. 10.1007/s11695-016-2415-6 PMC5339332 27783370

[25] Sickles EA (1998). Findings at mammographic screening on only one standard projection: outcomes analysis. Radiology.

[26] Raza S, Mackesy MM, Winkler NS (2016). Effect of training on qualitative mammographic density assessment. J Am Coll Radiol.

[27] Hawes D, Downey S, Pearce CL (2006). Dense breast stromal tissue shows greatly increased concentration of breast epithelium but no increase in its proliferative activity. Breast Cancer Res.

[28] Huo CW, Chew G, Hill P (2015). High mammographic density is associated with an increase in stromal collagen and immune cells within the mammary epithelium. Breast Cancer Res.

[29] Ghosh K, Brandt KR, Reynolds C (2012). Tissue composition of mammographically dense and non-dense breast tissue. Breast Cancer Res Treat.

[30] Woolcott CG, Cook LS, Courneya KS (2010). Mammographic density change with 1 year of aerobic exercise among postmenopausal women: a randomized controlled trial. Cancer Epidemiol Biomark Prev.

[31] Chen J, Pee D, Ayyagari R (2006). Projecting absolute invasive breast cancer risk in white women with a model that includes mammographic density. J Natl Cancer Inst.

[32] Brentnall AR, Harkness EF, Astley SM (2015). Mammographic density adds accuracy to both the Tyrer-Cuzick and Gail breast cancer risk models in a prospective UK screening cohort. Breast Cancer Res.

